# Supplementary material for: Joint processing of long- and short-read sequencing data with deep learning improves variant calling
Source: Cell Rep Methods. 2025 Jul 15;5(7):101107. doi: 10.1016/j.crmeth.2025.101107 (PMC12296420; doi:10.1016/j.crmeth.2025.101107)
Supplement: Document S2. Article plus supplemental information [file mmc9.pdf]

Cell Reports
Methods

Report

Joint processing of long- and short-read sequencing data with deep learning improves variant calling

Graphical abstract

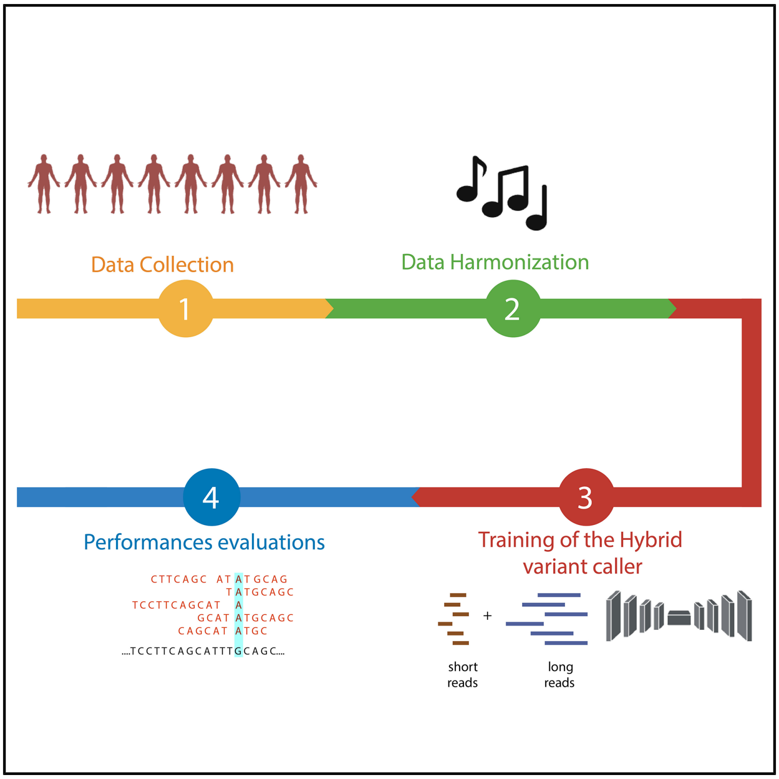

Authors

Gennaro Gambardella

Correspondence

gambardella@tigem.it

In brief

Gambardella shows that shallow short- and long-read sequencing combined with a retrained DeepVariant model improves small variant detection. This hybrid strategy offers a cost-effective alternative to deep single-tech sequencing and enables unified variant calling instead of post hoc merging across platforms.

Highlights

- Hybrid short- and long-read sequencing improves variant detection
- Joint modeling of hybrid inputs improves DeepVariant's performance
- Shallow hybrid sequencing yields competitive performance to deep single-tech sequencing
- Shallow hybrid sequencing may lower costs in large-scale clinical screening

Gambardella, 2025, Cell Reports Methods *5*, 101107
July 21, 2025 © 2025 The Author. Published by Elsevier Inc.
https://doi.org/10.1016/j.crmeth.2025.101107

CellPress

Cell Reports Methods

CellPress
OPEN ACCESS

Report

Joint processing of long- and short-read sequencing data with deep learning improves variant calling

Gennaro Gambardella[1,2,3,*]
[1]Telethon Institute of Genetics and Medicine, Naples, Italy
[2]Scuola Superiore Meridionale, Genomics and Experimental Medicine Program, Naples, Italy
[3]Lead contact
*Correspondence: gambardella@tigem.it
https://doi.org/10.1016/j.crmeth.2025.101107

MOTIVATION Short-read and long-read sequencing technologies offer distinct yet complementary strengths. Short reads excel at detecting small variants but struggle in complex or repetitive genome regions. In contrast, long reads provide better coverage of these regions and are well suited for identifying large structural variants, although their higher error rates can compromise the detection of small variants. Despite these complementary strengths, current variant-calling methods still rely on a single sequencing data type. As a result, there is an urgent need for hybrid approaches that can integrate both sequencing methods, leveraging their respective advantages to correct biases and improve overall detection accuracy.

SUMMARY

Despite the complementary strengths of short- and long-read sequencing approaches, variant-calling methods still rely on a single data type. In this study, we collected and harmonized Nanopore datasets of the seven healthy individuals in the GIAB project across three independent consortia. By leveraging these harmonized Nanopore data, we explore the benefits of using a hybrid DeepVariant model to jointly process Illumina and Nanopore data for germline variant detection. We show that a shallow hybrid long-short sequencing approach can match or surpass the germline variant detection accuracy of state-of-the-art single-technology methods, potentially reducing overall sequencing costs and enabling the detection of large germline structural variations. These findings hold great promise for molecular diagnostics in clinical settings, particularly for rare genetic disease screenings.

INTRODUCTION

Although individual rare diseases might seem insignificant, their cumulative effect is substantial. Current data suggest that between 3.5% and 5.9% of the global population, corresponding to roughly 300 million people, is affected by rare diseases.[1] Genetic factors are believed to cause approximately 80% of these rare conditions. Despite their widespread occurrence, most patients grappling with a rare or undiagnosed disease are limited to symptomatic relief, with a mere 5% having access to appropriate treatments. Accurate diagnosis is the cornerstone of effective handling of rare diseases. It enhances disease management and opens avenues for potential therapeutic interventions, all the while avoiding unnecessary treatments that could result in severe side effects.

Over the past decade, next-generation sequencing (NGS) has significantly enhanced our ability to identify the causative variant and understand the inheritance pattern of rare genetic conditions. Initiatives such as Care for Rare (https://www.care4rare.ca), Deciphering Developmental Disorders,[2] Rare and Undiagnosed Diseases Diagnostic Service,[3] and the Undiagnosed Diseases Network[4] have demonstrated the transformative impact of whole-exome (WES) and whole-genome (WGS) sequencing. However, it is crucial to acknowledge that only a fraction of patients (25%–35%) currently receive a definitive molecular diagnosis followed by actionable findings.

Difficulties in pinpointing the causative variant from WES/WGS data are often related to detection challenges where the causative variant is in a difficult-to-sequence region or is obscured due to biases in reference genomes and genomic datasets. To address this, long-read sequencing provides more accurate and complete information about the structure and function of the genome, especially for regions that are difficult to resolve with short-read sequencing, such as repetitive or low-complexity regions.

In summary, short-read NGS technologies excel at identifying small variants but struggle in complex regions. Conversely,

© 2025 The Author. Published by Elsevier Inc.
This is an open access article under the CC BY license (http://creativecommons.org/licenses/by/4.0/).

third-generation sequencing approaches based on long reads offer superior coverage of these regions and excel at detecting large structural variants but have a higher error rate (5%–15%) that can compromise small variant detection.[5] Indeed, even if advances like Oxford Nanopore Technology (ONT) have currently reduced the long-read sequencing costs of the human genome at 30× depth to about $850 per individual and its latest R10.4.1 chemistry can achieve a modal accuracy of Q20 for native reads, its high error rate compared to PacBio severely limits its use in clinical practice for identifying small variants that are crucial for diagnoses of genetic diseases.

Essentially, both short- and long-read sequencing offer complementary advantages. However, current variant-calling methods typically rely on a single data type, with limited attempts to combine PacBio and Illumina data.[6] Therefore, hybrid approaches that can synergize the advantages of both sequencing methods, correcting biases and enhancing accuracy, are urgently needed, especially for rare genetic diseases. Deep learning (DL) and artificial intelligence (AI) have recently emerged as powerful tools in data integration for several biological fields.[7] These technologies have the potential to correct errors and mitigate biases inherent in individual sequencing methods by combining their outputs in a synergistic framework with greater resolving power, thereby improving the accuracy of variant detection. In this context, convolutional neural networks (CNNs) have already been proven effective for variant-calling tasks due to their ability to handle complex DNA sequencing data. Indeed, one such CNN-based tool, DeepVariant,[8] developed by Google AI, effectively translates the problem of identifying variants into an image classification task, achieving high performance using short- or long-read data alone.

Here, to fully leverage the potential of both short-read and long-read sequencing, we developed a harmonized pipeline to process over 100 terabytes of publicly available raw ONT and Illumina sequencing data from the GIAB,[9] HPRC,[10] and ONTOD projects.[11] These data were then fed to DeepVariant to train a model to identify variants from hybrid short-long Nanopore-Illumina sequencing data.

We apply this hybrid model to several whole-genome case studies and show that it outperforms state-of-the-art variant callers that rely solely on one sequencing type. We show that our hybrid approach can improve the accuracy of small variant detection, including those located in challenging repetitive/low-complexity regions. We demonstrate that a hybrid shallow WGS strategy combining 15× ONT and 15× Illumina coverage can suffice to achieve high germline variant detection accuracy, offering a promising solution for integrated small and large variant detection in clinical settings at a lower overall cost compared to deep sequencing performed with a single technology.

RESULTS

A harmonized Nanopore dataset of individuals for training and benchmarking of variant-caller algorithms

Public consortia like the GIAB,[9] the HPRC,[10] and the ONTOD[11] projects provide valuable resources for developing and testing variant-calling methods. These projects offer high-quality Nanopore and Illumina sequencing data for a well-characterized cohort of seven healthy individuals (HG001–HG007), including two trio families.[9] Notably, the GIAB project includes a high-confidence set of "ground-truth" small germline variants for each individual established by merging results from over 10 different sequencing methodologies.[12,13] However, data from these projects are fragmented and processed with different pipelines, potentially introducing biases hindering their use for AI-based training.

To address these limitations, we built a harmonized Nanopore dataset specifically tailored for benchmarking variant-calling algorithms. We collected over 100 terabytes of raw sequencing data in FAST5/POD5 format from all seven individuals across the three consortia (Tables 1, S1, and S2). Data for Nanopore R10.4.1 chemistry were processed through a unified, publicly available pipeline we developed (https://github.com/gambalab/honey_pipes) and optimized for this specific data type. This pipeline handles the entire workflow from raw reads to aligned BAM files, ensuring consistency and avoiding biases (Figure S1; STAR Methods). The pipeline prioritizes simplex and duplex reads for precise allele frequency estimation, excluding redundant duplex parent reads (STAR Methods). For Nanopore R9.4.1 data, we converted FAST5 files to FASTQ using Guppy and aligned reads with minimap2 (Figure S1).

The Nanopore datasets processed with these workflows, including both aligned and not aligned sequencing data for each individual, are publicly available on the Sequence Read Archive repository from the National Center for Biotechnology Information (see the data and code availability section). This dataset offers a valuable resource for the scientific community to develop and test novel AI-driven variant-calling methods.

Jointly processing Nanopore-Illumina sequencing data enhances germline variant detection

Leveraging the harmonized data we generated, we investigated whether a hybrid sequencing approach based on short-read Illumina and long-read Nanopore data could improve small variant detection. To achieve this, we developed a custom DeepVariant[8] model tailored to jointly process short- and long-read sequences from hybrid Nanopore-Illumina aligned data (https://github.com/gambalab/honey_DeepVariant). We trained two DeepVariant models tailored to specific sequencing chemistries: (1) one adapted to recent Nanopore R10.4.1 chemistry and Illumina short-read data and (2) another adapted for previous Nanopore R9.4.1 chemistry and Illumina short-read data. We also employed a data augmentation strategy using downsampling (see STAR Methods) to increase the training dataset size, which resulted in over 200 million training examples for each hybrid model (Table S1). Excluding HG003, the true germline variants from chromosomes 1–19 of the other collected individuals were used for training, while variants from chromosome 21 of each individual were used to evaluate model performance on unseen data during the training stage.

Next, to evaluate our hybrid model's performance, we applied it to a series of case studies where ground-truth mutations were available from both GIAB consortia and ULTIMA genomics[14] and then compared our model's accuracy to state-of-the-art variant callers that work on a single sequencing technology. Specifically, to explore the synergistic effect of combining short

Table 1. Overview of Nanopore sequencing samples: Chemistry, coverage, source, and training utilization

Chemistry	Individual	Sample name	Read type	Avg. coverage (chr1–22,X,Y)	Used for training	Source	Data type	Basecaller
R9.4.1	HG001	HG001_ULR	ULR	43×	Y	GIAB	FAST5	Guppy 6
R9.4.1	HG002	HG002_ULR	ULR	30×	Y	GIAB	FAST5	Guppy 6
R9.4.1	HG002	HG002_LOR	LR	32×	Y	GIAB	FAST5	Guppy 6
R9.4.1	HG003	HG003_LOR	LR	28×	N	GIAB	FAST5	Guppy 6
R9.4.1	HG004	HG004_LOR	LR	27×	Y	GIAB	FAST5	Guppy 6
R9.4.1	HG005	HG005_ULR	ULR	40×	Y	GIAB	FAST5	Guppy 6
R9.4.1	HG005	HG005_LOR	LR	13×	Y	GIAB	FAST5	Guppy 6
R9.4.1	HG006	HG006_LOR	LR	13×	Y	GIAB	FAST5	Guppy 6
R9.4.1	HG007	HG007_LOR	LR	12×	Y	GIAB	FAST5	Guppy 6
R10.4.1	HG001	HG001_LOR_S01	LR	39×	Y	HPCR	FAST5	Dorado 0.7
R10.4.1	HG001	HG001_LOR_S02	LR	51×	Y	ONODR	POD5	Dorado 0.7
R10.4.1	HG002	HG002_ULR_S01	ULR	107×	Y	ONODR	FAST5	Dorado 0.7
R10.4.1	HG002	HG002_ULR_S02	ULR	53×	Y	HPCR	FAST5	Dorado 0.7
R10.4.1	HG002	HG002_LOR_S01	LR	54×	Y	HPCR	FAST5	Dorado 0.7
R10.4.1	HG002	HG002_LOR_S02	LR	116×	Y	HPCR	FAST5	Dorado 0.7
R10.4.1	HG003	HG003_LOR_S01	LR	45×	N	ONODR	POD5	Dorado 0.7
R10.4.1	HG003	HG003_LOR_S02	LR	65×	N	ONODR	POD5	Dorado 0.7
R10.4.1	HG004	HG004_LOR_S01	LR	38×	Y	HPCR	FAST5	Dorado 0.7
R10.4.1	HG004	HG004_LOR_S02	LR	53×	Y	HPCR	FAST5	Dorado 0.7
R10.4.1	HG005	HG005_LOR_S01	LR	61×	Y	HPCR	FAST5	Dorado 0.7

The table summarizes the Nanopore sequencing datasets used for the hybrid model development, detailing individual samples, read types, average coverage across chromosomes 1–22, X, and Y, their usage for training, sequencing source, data format, and basecaller version. The table distinguishes between R9.4.1 and R10.4.1 chemistries and highlights which samples contributed to training. Y, yes; N, no.

Illumina and long Nanopore reads, we simulated three WGS scenarios for individual HG003. In the first scenario, HG003 was sequenced at a hybrid depth of 50×, combining 30× Nanopore and 20× Illumina reads. The second scenario used 30× Nanopore sequencing only, while the third used 20× Illumina sequencing only. As shown in Figures 1A–1D and Tables S3 and S4, for single-nucleotide variant (SNV) detection, our hybrid model consistently outperforms leading Nanopore-only (DeepVariant,[8] Clair3,[15] and Nanocaller[16]) and Illumina-only (GATK4[17] and Strelka2[18]) variant callers in terms of the F1 score, true positive, false positive (FP), and false negative rates, regardless of the Nanopore chemistry used and independently of the ground-truth mutation dataset used. For small insertion or deletion (indel) detection (Figures 2A–2D; Tables S3 and S4), we observed a synergistic effect only when the latest Nanopore chemistry was used, especially when performances were evaluated on the ULTIMA genomics ground-truth mutation dataset. With previous Nanopore R9.4.1 chemistry, our hybrid model significantly outperformed Nanopore-only sequencing and performed comparably to Illumina-only variant callers, suggesting that in this case, the model is not able to synergistically use both types of reads, probably due to the higher error rate of R9.4.1 chemistry compared to R10.4.1 chemistry.

Next, we asked if our hybrid model's improved performance in detecting germline small variants could be extended to challenging genomic regions. To this end, following GIAB guidelines,[19] we stratified the genome of individual HG003 into seven challenging areas: tandem repeat regions (TRRs), low-mappability regions (LMRs), GC-enriched regions, regions enriched by variants with excess heterozygosity (i.e., which are defined by the InbreedingCoeff from the gnomAD database),[20] regions with long segmental duplications characterized by high sequence identity,[21] and collapsed duplication FP regions and population copy-number variation (CNV) FP regions (related to collapsed duplication errors in GRCh38, recently identified and corrected by the T2T consortium[20]). As shown in Figures 1E, 1F, 2E, and 2F and Tables S3 and S4, we found that, regardless of the type of small variant or Nanopore chemistry used, our hybrid model either outperformed or almost matched the sequencing technology best suited for these challenging regions, showing the model's ability to autonomously learn which sequencing technology to rely on for optimal variant detection in these regions.

Since the mutations of individual HG002 (the son of HG003) were included in the training, approximately 50% of HG003's variants were indirectly used during the training of our method, as well as for the Clair3, DeepVariant, and Nanocaller methods. To remove this data leakage, all validation metrics reported for HG003 were computed after removing variants shared with HG002 from both the ground truth and the predictions of each tool.

Optimizing shallow hybrid sequencing for accurate and cost-effective variant detection

Overall, these analyses demonstrated that combining sequencing data from R10.4.1 Nanopore chemistry and Illumina can improve

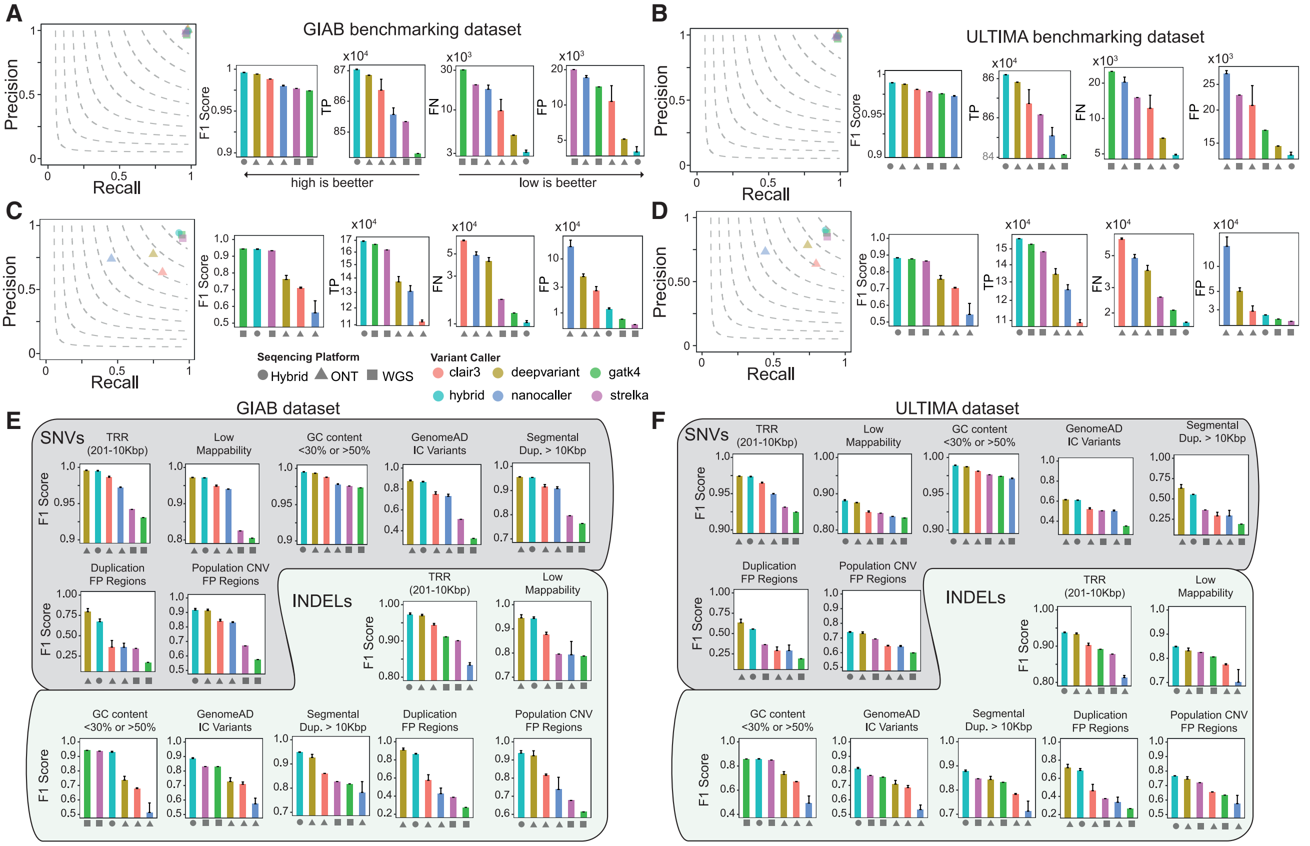

Figure 1. Combined Illumina and Nanopore R10.4.1 chemistry reads enhance germline small variant detection

(A) Comparisons between DeepVariant Hybrid model R10.4.1 and the state-of-the-art variant callers that rely on a single sequencing platform for the detection of germline SNVs in individual HG003 using the GIAB ground-truth mutation set. The hybrid model was tested on HG003, a mixture of 20× Illumina and 30× Nanopore reads. Single-platform callers (Nanopore only or Illumina only) used 30× Nanopore or 20× Illumina reads. TP, true positives; FN, false negatives; FP, false positives.

(B) Same as (A) but using ULTIMA as the ground-truth mutation set.

(C) Same as (A) but for indels.

(D) Same as (B) but for indels.

(E) Comparisons between DeepVariant Hybrid model R10.4.1 and the state-of-the-art variant callers that rely on a single sequencing platform for the detection of germline SNVs in individual HG003 stratified by difficult genome regions. Performances were measured using the GIAB ground-truth mutation set.

(F) Same as (E) but using the ULTIMA ground-truth mutation set.

In (A)–(F), data are presented as mean + standard deviation. For (A) and (B), the y axes for TP, FN, and FP are displayed on a $\log_{10}$ scale.

the accuracy of germline small variant detection, including those located in challenging repetitive or low-complexity genome regions. We next asked whether we could identify the most cost-effective shallow hybrid sequencing protocol with an optimal trade-off between sequencing depth and variant detection accuracy. For this purpose, we used all available samples from individual HG002 and generated several versions of chromosome 20 featuring different total hybrid coverage depths ranging from 10× to 35× (Figures 3A and 3B). We used chromosome 20 from individual HG002 due to the availability of the highest number of replicates for this individual. For these tests, we used the set of mutations provided by GIAB as the ground truth. As shown in Figures 3A and 3B, after a total hybrid coverage depth of 25–30×, the increase in the F1 score of variant detection becomes modest (Tables S5 and S6), indicating that this depth could represent a good trade-off between sequencing depth and variant detection accuracy. However, the SNV detection accuracy was independent of the small and long reads ratio only with R10.4.1 chemistry. In all other scenarios, the optimal trade-off was achieved within a range of 15–20× Illumina short reads and 10–15× Nanopore long reads (Figures 3A and 3B), indicating that while the two sequencing technologies can complement each other, the model has learned to prioritize short Illumina reads in these scenarios.

Finally, to further demonstrate the shallow hybrid sequencing approach's detection accuracy, we compared DeepVariant models trained on hybrid sequencing data versus those trained on individual sequencing data. Specifically, for each of the seven GIAB individuals, we trained three distinct models: one using hybrid sequencing data, one using Illumina-only data, and one using Nanopore-only data. Each model was trained on chromosome 1, while variants from chromosome 21 were used as validation

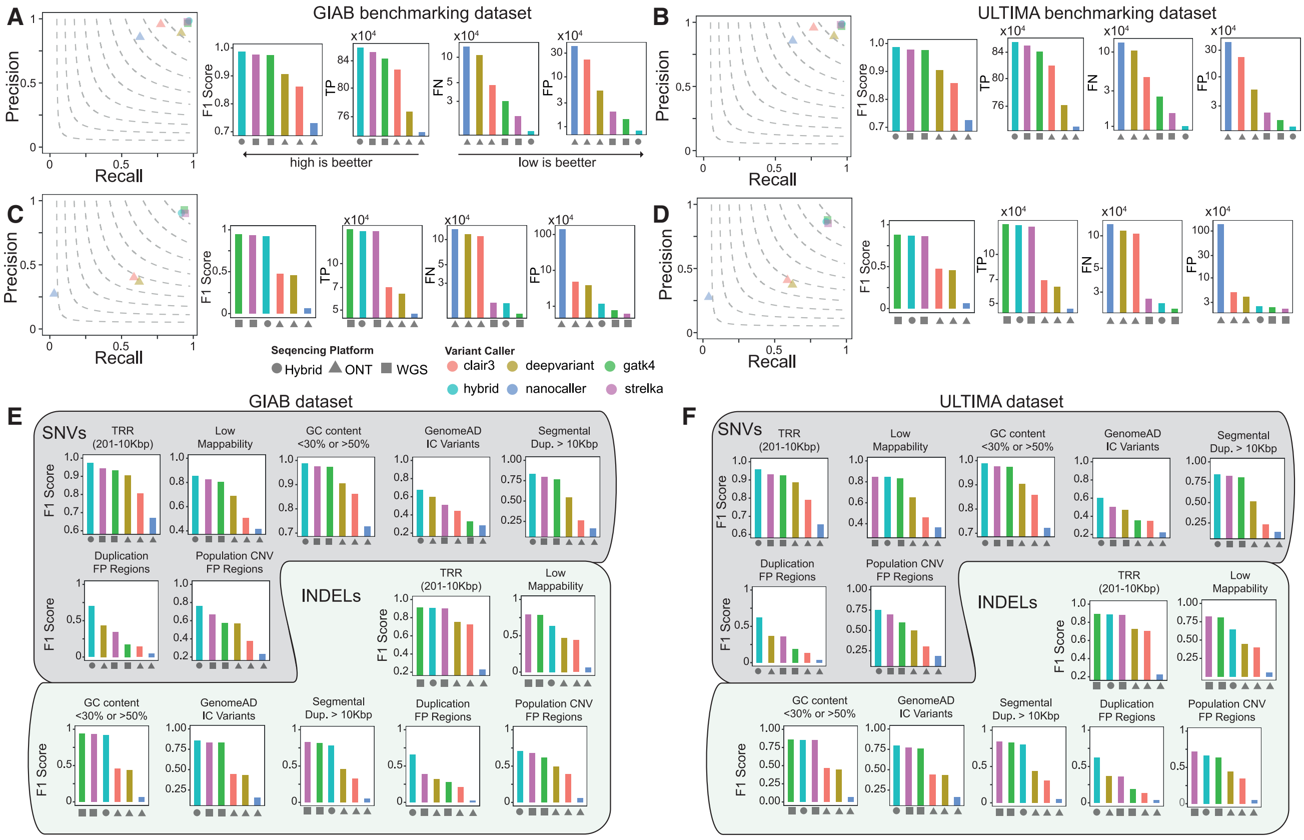

Figure 2. Combined Illumina and Nanopore R9.4.1 chemistry reads enhance germline small variant detection

(A) Comparisons between DeepVariant Hybrid model R9.4.1 and the state-of-the-art variant callers that rely on a single sequencing platform for the detection of germline SNVs in individual HG003 using the GIAB ground-truth mutation set. The hybrid model was tested on HG003, a mixture of 20× Illumina and 30× Nanopore reads. Single-platform callers (Nanopore only or Illumina only) used 30× Nanopore or 20× Illumina reads. TP, true positives; FN, false negatives; FP, false positives.

(B) Same as (A) but using ULTIMA as the ground-truth mutation set.

(C) Same as (A) but for indels.

(D) Same as (B) but for indels.

(E) Comparisons between DeepVariant Hybrid model R9.4.1 and the state-of-the-art variant callers that rely on a single sequencing platform for the detection of germline SNVs in individual HG003 stratified by difficult genome regions. Performances are measured using the GIAB ground-truth mutation set.

(F) Same as (E) but using the ULTIMA ground-truth mutation set.

data to assess performance on unseen data during training. Model performance was then evaluated based on variant detection on chromosome 21 (STAR Methods). All chromosomes were downsampled to a fixed total coverage of 30× (i.e., in the case of the hybrid model, we used 15× short and 15× long reads) using Nanopore data from the latest R10.4 chemistry. As shown in Figure S2, our results confirm the synergistic advantage of combining short and long reads for SNV detection. For indel detection, the hybrid model outperformed the Nanopore-only model while closely matching the performance of the Illumina-only model.

An advantage of long reads over short reads is their intrinsic ability to detect large structural variations (SVs). Therefore, we asked whether 10–15× of Nanopore long-read coverage would also be adequate for accurate SV detection. To test this, we used all available samples of individual HG002 for which ground-truth SVs were available from GIAB and generated several downsampled versions of this individual with a coverage depth ranging from 5× to 30× (see STAR Methods). We then employed state-of-the-art AI-driven SV callers, such as dysgu and SVision,[22,23] and assessed their performance as a function of Nanopore sequencing depth. As shown in Figure 2C, SVision consistently outperformed dysgu, but for both methods, the increase in the F1 score beyond an average depth of 15× was modest (Table S7), indicating that this depth of coverage is probably also sufficient for reliable structural variant identification from Nanopore long reads.

DISCUSSION

This study demonstrates the value of harmonized datasets and hybrid sequencing approaches for improved variant detection. We created a comprehensive resource by collecting and processing raw Nanopore data from three consortia (STAR Methods). We also developed state-of-the-art, user-friendly

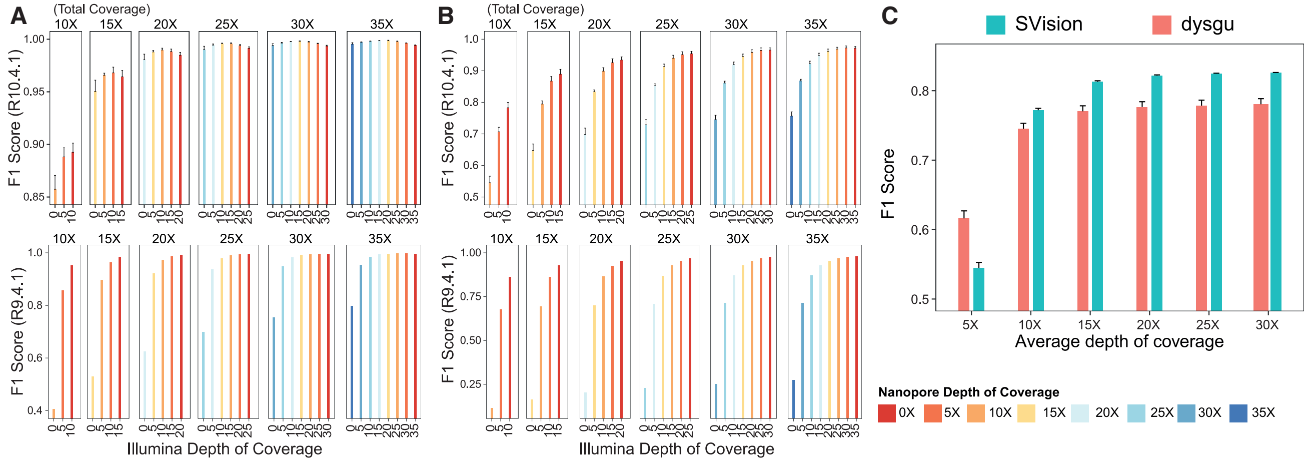

Figure 3. A shallow hybrid Nanopore-Illumina strategy for affordable whole-genome sequencing

(A) How a different ratio of Illumina and Nanopore reads affects the performance of the hybrid model for detecting germline SNVs. Tests were performed on chromosome 20 of the HG002 individual.

(B) Same as (A) but for germline indels.

(C) Performance of dysgu and SVision tools in identifying large SVs as a function of coverage depth of the whole genome of the HG002 individual sequenced with Nanopore technology chemistry R10.4.1.

In (A)–(C), data are presented as means + standard deviation.

pipelines for processing Illumina and Nanopore data, which are accessible through a single Docker/Singularity container.

Leveraging this harmonized dataset, we trained custom DeepVariant models to analyze short-read Illumina and long-read Nanopore data jointly. Our method enables the generation of unified call sets rather than merging variant calls from different technologies. Our findings suggest that shallow hybrid sequencing (about 15× coverage for both platforms) achieves F1 scores of 0.9980 and 0.9454 in detecting SNVs and indels, respectively. By contrast, structural variant identification with a coverage depth of 15× Nanopore long reads had an F1 score equal to 0.8129 with SVision (with 30×, the F1 score was equal to 0.8259).

Although the per-sample cost of a hybrid sequencing approach with 30× coverage equally distributed between Illumina and Nanopore platforms can vary depending on protocols, it offers potential cost savings over standard 30× Illumina WGS, especially in large-scale screening. However, our shallow hybrid approach always provides the advantage of detecting small and large SVs. Additionally, while not demonstrated in this study, Nanopore sequencing inherently enables whole-genome methylation profiling, further extending its utility.

Limitations of the study

While this study demonstrates the benefits of jointly processing short- and long-read sequencing data, several limitations should be considered. First, at present, this approach requires access to two distinct sequencing platforms (e.g., Illumina and Nanopore), each involving separate library preparation protocols and higher input DNA, which may limit its applicability in certain clinical or low-input settings. Second, although a joint short- and long-read approach can improve variant detection in complex genomic regions, our model was evaluated on a limited number of well-characterized reference genomes; therefore, its generalizability to genetically diverse or clinically relevant cohorts remains to be validated. Third, our strategy adapts an existing single-technology variant caller rather than implementing a fully native hybrid-aware algorithm; as such, future models explicitly designed for hybrid input data may further enhance the performance. Finally, while structural variant detection was assessed using state-of-the-art tools, we did not develop or benchmark a unified method for joint small and structural variant calling from hybrid data that may further show the benefits of using hybrid sequencing approaches.

RESOURCE AVAILABILITY

Lead contact

Further information and requests for resources and reagents should be directed to and will be fulfilled by the lead contact, Gennaro Gambardella (gambardella@tigem.it).

Materials availability

This study did not generate new unique materials.

Data and code availability

- Harmonized unaligned and aligned Nanopore data have been deposited on SRA with accession codes PRJNA1191200 for R10.4 chemistry and PRJNA1193572 for R9.4 chemistry.
- Pipelines to process both short and long reads are available at https://github.com/gambalab/honey_pipes. Our custom hybrid DeepVariant tool is available at https://github.com/gambalab/honey_DeepVariant. Archival versions are included in the key resources table.
- Any additional information required to reanalyze the data reported in this paper is available from the lead contact upon request.

ACKNOWLEDGMENTS

This work was supported by the Telethon Foundation, the Italian Ministry of Health (Piano Operativo Salute Traiettoria 3, T3-AN-09, “Genomed”), the Italian Ministry of University and Research, and the European Union (Next

Generation EU-MUR-PRIN-2022, Project PNC 0000001 D3 4 Health). We also express our gratitude to the TIGEM IT core and CINECA for providing computational support through their cluster infrastructure and to Cathal Wilson for his kindness and meticulous manuscript proofreading.

AUTHOR CONTRIBUTIONS

G.G. performed all bioinformatics analyses, wrote the manuscript, and conceived the original idea.

DECLARATION OF INTERESTS

The author declares no competing interests.

DECLARATION OF GENERATIVE AI AND AI-ASSISTED TECHNOLOGIES IN THE WRITING PROCESS

During the preparation of this work, the author used ChatGPT and Gemini to proofread the manuscript. After using this tool/service, the author reviewed and edited the content as needed and takes full responsibility for the publication's content.

STAR★METHODS

Detailed methods are provided in the online version of this paper and include the following:

- KEY RESOURCES TABLE
- EXPERIMENTAL MODEL AND STUDY PARTICIPANT DETAILS
- METHOD DETAILS
  - Nanopore R9.4.1 chemistry data collection and processing
  - Nanopore R10.4.1 chemistry data collection and processing
  - Short illumina reads data collection and processing
  - Training hybrid custom DeepVariant models
  - Small variant callers comparisons on individual HG003 (Figures 1 and 2)
  - Identification of the best ratio of long and short reads to enable shallow hybrid sequencing (Figure 3)
  - DeepVariant model comparisons (Figure S2)
  - Large structural variant caller evaluation
  - GIAB samples
- QUANTIFICATION AND STATISTICAL ANALYSIS

SUPPLEMENTAL INFORMATION

Supplemental information can be found online at https://doi.org/10.1016/j.crmeth.2025.101107.

Received: November 21, 2024
Revised: March 15, 2025
Accepted: June 19, 2025
Published: July 15, 2025

REFERENCES

1. Nguengang Wakap, S., Lambert, D.M., Olry, A., Rodwell, C., Gueydan, C., Lanneau, V., Murphy, D., Le Cam, Y., and Rath, A. (2020). Estimating cumulative point prevalence of rare diseases: analysis of the Orphanet database. Eur. J. Hum. Genet. *28*, 165–173.
2. Firth, H.V., and Wright, C.F.; DDD Study (2011). The Deciphering Developmental Disorders (DDD) study. Dev. Med. Child Neurol. *53*, 702–703.
3. Baynam, G., Pachter, N., McKenzie, F., Townshend, S., Slee, J., Kiraly-Borri, C., Vasudevan, A., Hawkins, A., Broley, S., Schofield, L., et al. (2016). The rare and undiagnosed diseases diagnostic service – application of massively parallel sequencing in a state-wide clinical service. Orphanet J. Rare Dis. *11*, 77.
4. Gahl, W.A., Wise, A.L., and Ashley, E.A. (2015). The Undiagnosed Diseases Network of the National Institutes of Health: A National Extension. JAMA *314*, 1797–1798.
5. Logsdon, G.A., Vollger, M.R., and Eichler, E.E. (2020). Long-read human genome sequencing and its applications. Nat. Rev. Genet. *21*, 597–614.
6. Ramachandran, A., Lumetta, S.S., Klee, E.W., and Chen, D. (2021). HELLO: improved neural network architectures and methodologies for small variant calling. BMC Bioinf. *22*, 404.
7. Erfanian, N., Heydari, A.A., Feriz, A.M., Iañez, P., Derakhshani, A., Ghasemigol, M., Farahpour, M., Razavi, S.M., Nasseri, S., Safarpour, H., and Sahebkar, A. (2023). Deep learning applications in single-cell genomics and transcriptomics data analysis. Biomed. Pharmacother. *165*, 115077.
8. Poplin, R., Chang, P.C., Alexander, D., Schwartz, S., Colthurst, T., Ku, A., Newburger, D., Dijamco, J., Nguyen, N., Afshar, P.T., et al. (2018). A universal SNP and small-indel variant caller using deep neural networks. Nat. Biotechnol. *36*, 983–987.
9. Zook, J.M., Catoe, D., McDaniel, J., Vang, L., Spies, N., Sidow, A., Weng, Z., Liu, Y., Mason, C.E., Alexander, N., et al. (2016). Extensive sequencing of seven human genomes to characterize benchmark reference materials. Sci. Data *3*, 160025.
10. Liao, W.-W., Asri, M., Ebler, J., Doerr, D., Haukness, M., Hickey, G., Lu, S., Lucas, J.K., Monlong, J., Abel, H.J., et al. (2023). A draft human pangenome reference. Nature *617*, 312–324.
11. Oxford Nanopore Technologies Open Data. Oxford Nanopore Technologies Benchmark Datasets. https://registry.opendata.aws/ont-open-data/.
12. Zook, J.M., McDaniel, J., Olson, N.D., Wagner, J., Parikh, H., Heaton, H., Irvine, S.A., Trigg, L., Truty, R., McLean, C.Y., et al. (2019). An open resource for accurately benchmarking small variant and reference calls. Nat. Biotechnol. *37*, 561–566.
13. Krusche, P., Trigg, L., Boutros, P.C., Mason, C.E., De La Vega, F.M., Moore, B.L., Gonzalez-Porta, M., Eberle, M.A., Tezak, Z., Lababidi, S., et al. (2019). Best practices for benchmarking germline small-variant calls in human genomes. Nat. Biotechnol. *37*, 555–560.
14. Almogy, G., Pratt, M., Oberstrass, F., Lee, L., Mazur, D., Beckett, N., Barad, O., Soifer, I., Perelman, E., Etzioni, Y., et al. (2022). Cost-efficient whole genome-sequencing using novel mostly natural sequencing-by-synthesis chemistry and open fluidics platform. bioRxiv. https://doi.org/10.1101/2022.05.29.493900.
15. Zheng, Z., Li, S., Su, J., Leung, A.W.S., Lam, T.W., and Luo, R. (2022). Symphonizing pileup and full-alignment for deep learning-based long-read variant calling. Nat. Comput. Sci. *2*, 797–803.
16. Ahsan, M.U., Liu, Q., Fang, L., and Wang, K. (2021). NanoCaller for accurate detection of SNPs and indels in difficult-to-map regions from long-read sequencing by haplotype-aware deep neural networks. Genome Biol. *22*, 261.
17. Poplin, R., Ruano-Rubio, V., DePristo, M.A., Fennell, T.J., Carneiro, M.O., Van der Auwera, G.A., Kling, D.E., Gauthier, L.D., Levy-Moonshine, A., Roazen, D., et al. (2018). Scaling accurate genetic variant discovery to tens of thousands of samples. Preprint at bioRxiv. https://doi.org/10.1101/201178.
18. Kim, S., Scheffler, K., Halpern, A.L., Bekritsky, M.A., Noh, E., Källberg, M., Chen, X., Kim, Y., Beyter, D., Krusche, P., and Saunders, C.T. (2018). Strelka2: fast and accurate calling of germline and somatic variants. Nat. Methods *15*, 591–594.
19. Dwarshuis, N., Kalra, D., McDaniel, J., Sanio, P., Jerez, P.A., Jadhav, B., Huang, W.E., Mondal, R., Busby, B., Olson, N.D., et al. (2023). The GIAB genomic stratifications resource for human reference genomes. Preprint at bioRxiv. https://doi.org/10.1101/2023.10.27.563846.
20. Aganezov, S., Yan, S.M., Soto, D.C., Kirsche, M., Zarate, S., Avdeyev, P., Taylor, D.J., Shafin, K., Shumate, A., Xiao, C., et al. (2022). A complete

reference genome improves analysis of human genetic variation. Science *376*, eabl3533.

21. Prodanov, T., and Bansal, V. (2020). Sensitive alignment using paralogous sequence variants improves long-read mapping and variant calling in segmental duplications. Nucleic Acids Res. *48*, e114.
22. Cleal, K., and Baird, D.M. (2022). Dysgu: efficient structural variant calling using short or long reads. Nucleic Acids Res. *50*, e53.
23. Lin, J., Wang, S., Audano, P.A., Meng, D., Flores, J.I., Kosters, W., Yang, X., Jia, P., Marschall, T., Beck, C.R., and Ye, K. (2022). SVision: a deep learning approach to resolve complex structural variants. Nat. Methods *19*, 1230–1233.
24. Shafin, K., Pesout, T., Chang, P.C., Nattestad, M., Kolesnikov, A., Goel, S., Baid, G., Kolmogorov, M., Eizenga, J.M., Miga, K.H., et al. (2021). Haplotype-aware variant calling with PEPPER-Margin-DeepVariant enables high accuracy in nanopore long-reads. Nat. Methods *18*, 1322–1332.
25. Danecek, P., Bonfield, J.K., Liddle, J., Marshall, J., Ohan, V., Pollard, M.O., Whitwham, A., Keane, T., McCarthy, S.A., Davies, R.M., and Li, H. (2021). Twelve years of SAMtools and BCFtools. GigaScience *10*, giab008.
26. Li, H. (2018). Minimap2: pairwise alignment for nucleotide sequences. Bioinformatics *34*, 3094–3100.
27. English, A.C., Menon, V.K., Gibbs, R.A., Metcalf, G.A., and Sedlazeck, F.J. (2022). Truvari: refined structural variant comparison preserves allelic diversity. Genome Biol. *23*, 271.
28. Tarasov, A., Vilella, A.J., Cuppen, E., Nijman, I.J., and Prins, P. (2015). Sambamba: fast processing of NGS alignment formats. Bioinformatics *31*, 2032–2034.

STAR★METHODS

KEY RESOURCES TABLE

REAGENT or RESOURCE	SOURCE	IDENTIFIER
Deposited data		
Small Variants benchmarks v4.2.1	GIAB consortium	https://ftp-trace.ncbi.nlm.nih.gov/ReferenceSamples/giab/release/
Structural Variants benchmarks v1.1	GIAB consortium	https://ftp-trace.ncbi.nlm.nih.gov/ReferenceSamples/giab/data/AshkenazimTrio/analysis/NIST_HG002_DraftBenchmark_defrabbV0.019-20241113/
Harmonized nanopore Data flowcell R10.4.1	This manuscript	https://www.ncbi.nlm.nih.gov/bioproject/PRJNA1191200
Harmonized nanopore Data flowcell R9.4.1	This Manuscript	https://www.ncbi.nlm.nih.gov/bioproject/PRJNA1193572
Software and algorithms		
DeepVariant (v1.6.1)	Poplin et al.[8]	https://github.com/google/DeepVariant
PEPPER-DeepVariant (v0.8)	Shafin et al.[24]	https://github.com/kishwarshafin/pepper
Clair3 (v1.0.11)	Zheng et al.[15]	https://github.com/HKU-BAL/Clair3
Nanocaller (v3.6.0)	Ahsan et al.[16]	https://github.com/WGLab/NanoCaller
HaplotypeCaller (GATK v4.4.0.0)	Poplin et al.[17]	https://github.com/broadinstitute/gatk
Strelka2 (v2.9.9)	Kim et al.[18]	https://github.com/Illumina/strelka
Samtools (v1.21)	Petr et al.[25]	https://github.com/samtools/samtools
Dorado (v0.7.2)	Oxford Nanopore Technology.	https://github.com/nanoporetech/dorado
Guppy (v6.0)	Oxford Nanopore Technology.	https://nanoporetech.com/software/other/guppy
Minimap2 (v2.28)	Heng et al.[26]	https://github.com/lh3/minimap2
DRAGMAP (v1.3.2)	This Manuscript	https://github.com/gambalab/DRAGMAP https://doi.org/10.5281/zenodo.15595504
SVision (v1.4)	Lin et al.[23]	https://github.com/xjtu-omics/SVision
Dysgu (1.6.6)	Cleal et al.[22]	https://github.com/kcleal/dysgu
Hap.py (v00.3.12)	Krusche et al.[13]	https://github.com/Illumina/hap.py
Honey_pipes	This manuscript	https://github.com/gambalab/honey_pipes https://doi.org/10.5281/zenodo.15595485
Honey_DeepVariant	This Manuscript	https://github.com/gambalab/honey_DeepVariant https://doi.org/10.5281/zenodo.15595448
Truvari (v4.3.1)	Adam et al.[27]	https://github.com/ACEnglish/truvari
Sambamba (v1.0.1)	Tarasov et al.[28]	https://github.com/biod/sambamba

EXPERIMENTAL MODEL AND STUDY PARTICIPANT DETAILS

This study analyzed publicly available sequencing data and did not utilize new biological samples. The sequencing data utilized from GIAB were from healthy donors. HG004 and HG007 are from female individuals, while the remaining five samples are from male individuals.

METHOD DETAILS

Nanopore R9.4.1 chemistry data collection and processing

We collected Nanopore sequencing data for all seven individuals (HG001-HG007) in FAST5 format from the GIAB consortium (links provided in Table S1). We collected a total of nine samples, as HG002 and HG005 were sequenced with both long and ultra-long read approaches (Table S1). Base calling was performed using *Guppy v6* with the *super accurate* model, generating FASTQ files. Guppy was either run on our GPUs cluster equipped with 4 Nvidia A40 or Leonardo CINECA supercomputer equipped with custom Nvidia A100 GPUs. Alignment to the reference genome HG38 (primary assembly from GENCODE) was achieved using *minimap2* with the *map-ont* preset alias.

Nanopore R10.4.1 chemistry data collection and processing

Nanopore R10.4.1 FAST5/POD5 data for individuals HG001-HG005 were collected from HPRC and ONTOD consortia (links in Table S1). We collected a total of 11 samples, as HG001, HG003, and HG004 were sequenced with both long and ultra-long read approaches, while HG002 was sequenced in quadruplicate, twice using long reads and twice using the ultra-long read approach (Table S1). When starting with FAST5 format, the data was first converted to POD5 with the *pod5* Python library. Subsequently, POD5 files were split by channels for optimized base calling. Split POD5 files were base called with *Dorado v0.7.2* using the *super accurate* model in duplex mode, generating unaligned BAM files. We preferred BAM over FASTQ due to its inclusion of read type information (simplex, duplex, or parent), crucial for accurate allele frequency estimation. Since FASTQ lacks this information, distinguishing read types is only possible with BAM input generated by the Dorado duplex base calling command. To ensure precise allele frequency estimation, we strictly aligned only simplex and duplex reads, excluding redundant duplex parent reads that could distort variant calling. Read selection was performed with *samtools*. Dorado was run either on our GPUs cluster equipped with 4 Nvidia A40 or Leonardo CINECA supercomputer equipped with custom Nvidia A100 GPUs. Alignment to the HG38 reference genome (primary assembly from GENCODE) was done using *minimap2* with the *lr:hqae* preset alias. Our pipelines for processing Nanopore data from FAST5/POD5 to aligned BAM files are publicly available on GitHub (https://github.com/gambalab/honey_pipes). These pipelines are encoded into the three scripts *run_split_pod5.sh*, *run_dorado_duplex.sh* and *run_aln_long.sh*.

Short illumina reads data collection and processing

Illumina short-read FASTQ data for individuals HG001-HG007 was obtained from the National Institute of Standards and Technology (NIST) public FTP archive (ftp.ncbi.nlm.nih.gov). These data were used to generate hybrid short-long sequencing samples for training our DeepVariant hybrid model. Each flow cell offered an approximate 20X coverage depth. These FASTQ files were initially pre-processed using the *bbduk* tool (https://github.com/BioInfoTools/BBMap) to remove low-quality bases and adapter sequences. Subsequently, the pre-processed reads were aligned to the reference genome HG38 (primary assembly from GENCODE) using our custom DRAGMAP aligner, available at https://github.com/gambalab/DRAGMAP. Finally, duplicate reads were filtered out using *samtools markdup*. The complete processing pipeline is encapsulated within the run_aln_short.sh script, accessible within our Docker/Singularity container at https://github.com/gambalab/honey_pipes.

Training hybrid custom DeepVariant models

We created hybrid sequencing datasets to train two DeepVariant models tailored to specific nanopore chemistries. For each individual, Nanopore sequencing data (aligned BAM files) were merged with Illumina data (aligned BAM files) using *samtools merge*. One dataset combined R9.4.1 chemistry Nanopore data with Illumina reads, while the other used R10.4.1 chemistry Nanopore data. To increase training data diversity, downsampled versions of each hybrid sample were created using *sambamba*, containing 50% of both short and long reads. We followed the official DeepVariant training guidelines and utilized chromosomes 1–19 from all hybrid samples for training. Chromosome 21 was reserved for validation, mirroring the approach of the original DeepVariant manuscript.[8] Examples useful for training and validation were generated using Genome in a Bottle small variant true variants of each individual (v4.2.1), but only using the truth confident region of chromosomes 1–19 and 21. The training stage was employed using default DeepVariant channels 1–6 and was carried out on a machine equipped with Nvidia RTX 4090 and RTX 4080 Ti GPUs. After 10 epochs (approximately 11 days), training was stopped, and the best-performing checkpoint was saved. A batch size of 1,024 or 512 was used for R9.4.1 and R10.4.1 hybrid models, respectively. For the hybrid model R9.4.1, 258,665,415 examples were used for training and 3,455,573 for model validation during training. For the hybrid model R10.4.1, 210,962,694 examples were used for training and 2,584,574 for monitoring the performance of the model during training. Our custom DeepVariant tool, specifically designed for hybrid Oxford Nanopore technology analysis (named "honey_DeepVariant"), is publicly available on GitHub at https://github.com/gambalab/honey_DeepVariant.

Small variant callers comparisons on individual HG003 (Figures 1 and 2)

For Nanopore R10.4.1 chemistry, samples were downsampled to 30X coverage before processing with DeepVariant v1.6.1, Clair3, or Nanocaller. Downsampling was performed with the *sambamba* tool. Samples from R9.4.1 chemistry were used as they were, since they already had an average coverage depth of about 30X (Table S1). Since no DeepVariant model exists for R9.4.1 chemistry, we employed pepper-DeepVariant[24] as recommended by the DeepVariant authors (https://github.com/google/DeepVariant) to evaluate DeepVariant performance on chemistry R9.4.1. The two short-read variant callers (GATK4 and Strelka2) were assessed on Illumina data downsampled to 20X coverage (obtained from the GIAB consortium). Downsampling was performed with the *sambamba* tool. GATK4 followed best practices suggested by GATK, including Base Quality Score Recalibration around known SNPs and INDELs from the 1000 Genomes Project, while Strelka2 used standard parameters. GATK4 variant filtering was performed using the hard filtering strategy as per GATK guidelines (https://gatk.broadinstitute.org/hc/en-us/articles/360035531112–How-to-Filter-variants-either-with-VQSR-or-by-hard-filtering). To test the performance of the hybrid model BAM files, the average coverage of 50X was instead built by merging 30X from Nanopore and 20X from Illumina. Bam file merging was performed using *samtools*. Finally, the performance of all variant callers, including our custom hybrid variant caller, was measured using the hap.py tool (https://github.com/Illumina/hap.py) in accordance with GIAB guidelines.[12,13] We analyzed only *pass* mutations within confident regions for HG003, utilizing GIAB v4.2.1 small variant benchmarks or ULTIMA genomics variants from s3://ultimagen-feb-2024-giab/DeepVariant_vcfs.

Stratification files were obtained from the GIAB consortium's ftp server. To eliminate bias from training on HG002 (son of HG003), which includes about 50% of HG003's variants, we removed shared variants from HG003's ground truth and predictions before evaluating the performance of each tool. This corrected the leakage present in our method, Clair3, DeepVariant, and Nanocaller, which have all been trained using mutations of individual HG002.

Identification of the best ratio of long and short reads to enable shallow hybrid sequencing (Figure 3)

The different versions of chromosome 20 of individual HG002 for all chemistry R10.4, R9.04, and Illumina were built by first downsampling the original BAM file and then merging the obtained downsampled Illumina and Nanopore BAM files. The *sambamba* tool was used for downsampling, while the *samtool* was used for merging Illumina and Nanopore BAM files.

DeepVariant model comparisons (Figure S2)

For each of the seven GIAB individuals, three distinct DeepVariant models were trained: one using hybrid sequencing data, one using Illumina-only data, and one using Nanopore-only data. Each model was trained on chromosome 1, while variants from chromosome 21 were used as validation data to assess performance on unseen data during training. The models were trained with a maximum of 50 epochs and an early stopping condition of 10 epochs. Model performance was then evaluated on chromosome 21 of the same individual. For training and testing, all chromosomes were downsampled to a fixed total coverage of 30X (i.e., for the hybrid model, we used 15X short reads and 15X long reads). The Nanopore data used to train hybrid models were those from R10.4 chemistry. The ground truth mutation set used to assess model performance was the GIAB v4.2.1 small variant benchmarks.

Large structural variant caller evaluation

We evaluated the performance of two structural variant (SV) callers, Dysgu and SVision, on Nanopore R10.4.1 chemistry data for individual HG002. The four collected samples were downsampled to various depths of coverage (5X to 30X, in 5X increments) using the *sambamba* tool. Dysgu and SVision were then run on each downsampled dataset with standard parameters. GIAB guidelines were followed for performance evaluation using *truvari*.[27] The SV benchmarks v1.1 for HG38 of individual HG002, available from the GIAB project (https://www.nist.gov/programs-projects/genome-bottle), served as the truth set for this analysis.

GIAB samples

Raw sequencing data were generated from DNA isolated from human cell lines derived from healthy individuals. HG004 and HG007 are female, while the remaining five samples are from male individuals. According to NIST's Genome in a Bottle project (https://www.nist.gov/programs-projects/genome-bottle), data and analyses from short-read and long-read sequencing methods are publicly available without a publication embargo.

QUANTIFICATION AND STATISTICAL ANALYSIS

Small variant caller performances were estimated with happy tool and following GIAB guidelines[13] while performances of large structural variant callers were estimated using truvari[27] as indicated in the GIAB guidelines.

Cell Reports Methods, Volume *5*

Supplemental information

Joint processing of long- and short-read sequencing data with deep learning improves variant calling

Gennaro Gambardella

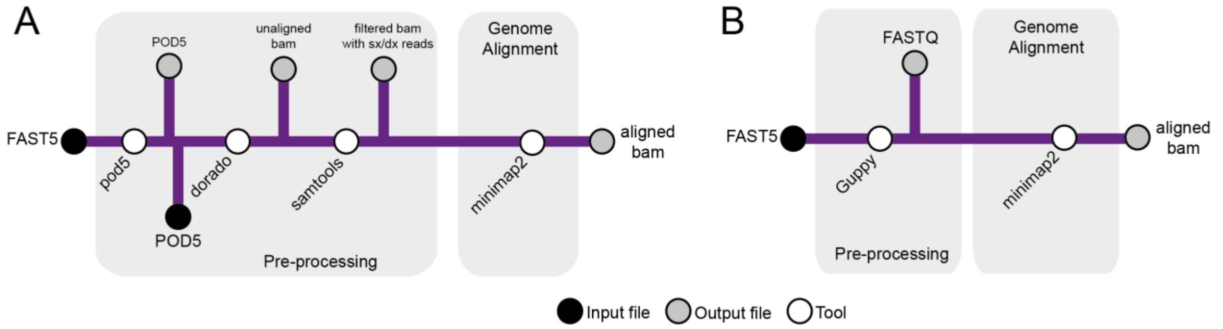

Figure S01 – Pipeline for harmonized Nanopore dataset. Related to Table 1. (**A**) For Nanopore R10.4.1 data, FAST5 files are converted to POD5, split by channels, and then processed with Dorado for duplex basecalling. Samtools is used to filter out redundant duplex parent reads, retaining simplex and duplex reads. Minimap2 aligns these reads to a reference genome. (**B**) For Nanopore R9.4.1 data, Guppy converts FAST5 files to FASTQ, and minimap2 aligns the long reads.

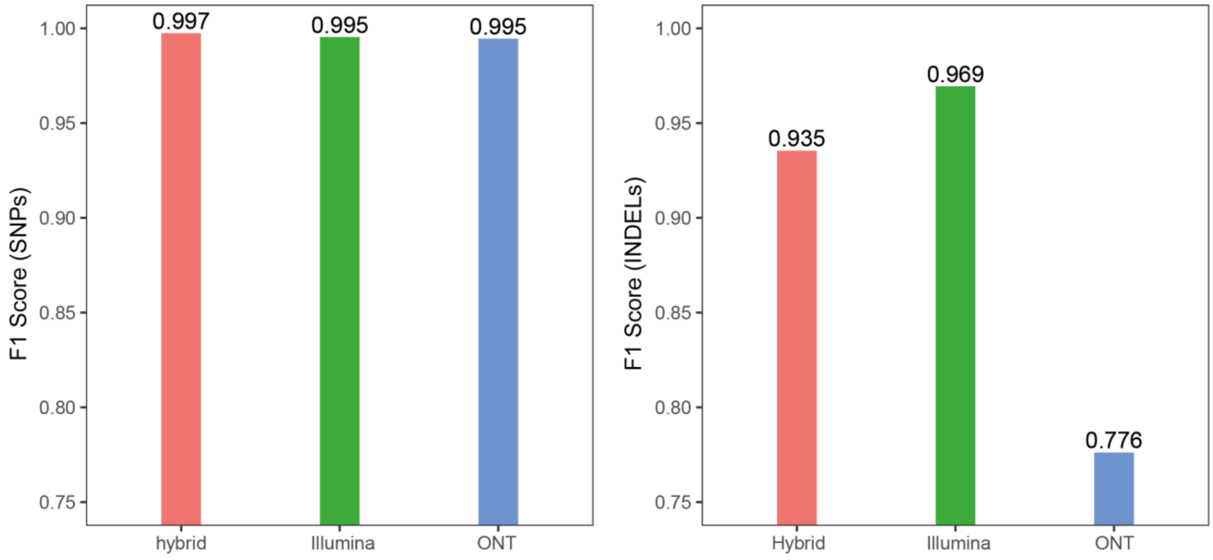

Figure S02 – DeepVariant model comparisons. Related to Figure 1. For each of the seven GIAB individuals, we trained three distinct DeepVariant models: one using hybrid sequencing data, one using Illumina-only data, and one using Nanopore-only data. Each model was trained on chromosome 1, while variants from chromosome 21 were used as validation data to assess performance on unseen data during training. Model performance was then evaluated based on variant detection on chromosome 21 of the same individual. All chromosomes were downsampled to a fixed total coverage of 30× (i.e., for the hybrid model, we used 15× short reads and 15× long reads) and analysed using Nanopore data from the latest R10.4 chemistry. (**A**) Average SNP detection performance of the three models on chromosome 21 across seven individuals. (**B**) Average INDEL detection performance of the three models on chromosome 21 across seven individuals. For both analyses, the ground truth mutation set was obtained from GIAB.
